# Influence of Tumor Subtype, Radiological Sign and Prognostic Factors on Tumor Size Discrepancies Between Digital Breast Tomosynthesis and Final Histology

**DOI:** 10.7759/cureus.6046

**Published:** 2019-10-31

**Authors:** Alessandro Garlaschi, Massimo Calabrese, Federico Zaottini, Simona Tosto, Marco Gipponi, Paola Baccini, Maurizio Gallo, Alberto Stefano Tagliafico

**Affiliations:** 1 Radiology, Ospedale Policlinico San Martino, Genova, ITA; 2 Radiology, University of Genoa, Genova, ITA; 3 Surgery, Ospedale Policlinico San Martino, Genova, ITA; 4 Pathology, University of Genova/ AOU IRCCS Policlinico San Martino, Genova, ITA; 5 Internal Medicine, University of Genoa, Genova, ITA; 6 Radiology, University of Genova/ AOU IRCCS Policlinico San Martino, Genova, ITA

**Keywords:** tumor subtype, radiological sign, prognostic factors, digital breast tomosynthesis, size discrepancies

## Abstract

Background

Influence of tumor subtype, radiological sign and prognostic factors on tumor size discrepancies between DBT and final histology has not been completely investigated so far.

Purpose

To study the influence of tumor subtype, radiological sign and prognostic factors on tumor size discrepancies between digital breast tomosynthesis and final histology.

Material and methods

This is a retrospective study conducted between January 2015 and December 2016. After IRB approval, 130 consecutive patients with breast cancer diagnosed with digital breast tomosynthesis (DBT) were evaluated. A discrepancy between DBT and final histology was considered present if the difference was above the cut-off of 5 mm. Tumor subtype, radiological sign and prognostic factors were evaluated in patients with discrepancies. Descriptive statistic and non-parametric tests were used.

Results

A total of 105 cases of cancer, in 96 patients, all female, were included. Mean age was 61 years (range: 35-82 yrs). In 19 (18.1%) cases, discrepancies were found: 13 (68.4%) were underestimated by DBT. For tumor subtype, 10 (52.6%) were infiltrating lobular carcinomas (ILC) (p < 0.01). Fourteen (73.7%) discordant cases were architectural distortions (p < 0.01). Prognostic factors did not affect tumor size discrepancies.

Conclusion

ILC or an architectural distortion represents the majority of cases of tumor size discrepancies between DBT and final histology.

## Introduction

Accurate size prediction of an invasive breast cancer is important in preoperative planning, as a prognostic indicator and consequently for patient management. With the increasing use of breast-conserving surgery and neoadjuvant chemotherapy, the ability to correctly determine maximum tumor extent non-invasively is essential. Under-estimating pre-surgical tumor size may lead to incomplete margins and consequently to re-excision [1]. Although pathologic measurement is regarded as the gold standard, important therapeutic decisions are made on the basis of the tumor size assessment by physical examination and imaging, indeed the pathological tumor staging could not be yet available [1-3].

There have been several attempts to predict breast tumor size based on medical imaging [1-5]. Preoperative tumor size is commonly measured on mammography and ultrasonography. However, using mammography tumor measurements are affected by superimposition of anatomical structures due to the 2D nature of mammography especially at the tumor boundaries [3-5]. Ultrasound, on the other hand, is likely to underestimate the real tumor size due to the overlap in the appearance among desmoplastic reaction and tumoral tissue [5]. Digital breast tomosynthesis, a pseudo-3D mammography, could overcome these limitations by reducing the effect of tissue superimposition [1-5]. Digital breast tomosynthesis (DBT) is a three-dimensional X-ray breast imaging method in which high spatial-resolution tomographic images of the breast are reconstructed from multiple low-dose projection exposures acquired by a digital detector from a mammographic X-ray source which moves over a limited angle. The total mean glandular dose for a DBT examination is comparable to that of a two-view mammographic examination, with a higher sensitivity for breast cancer detection [6,7]. DBT and magnetic resonance imaging (MRI) are considered superior to digital mammography and ultrasound (US) in the preoperative assessment of breast tumor size [1] and MRI overestimated tumor size in ductal cancers and slightly underestimated it in lobular cancers [2]. Moreover, DBT showed characteristic imaging features of breast cancers according to molecular subtype [8], but the influence of tumor subtype, radiological sign and prognostic factors on tumor size discrepancies between DBT and final histology has not been completely investigated so far. Therefore, the purpose of this study is to evaluate the influence of tumor subtype, radiological sign and prognostic factors on tumor size discrepancies between digital breast tomosynthesis and final histology.

## Materials and methods

IRB approval was obtained and written informed consent was waived (Blinded). This is a retrospective study, based on data already stored in the Hospital (Blinded) database. A total of 130 consecutive breast cancer patients (from January 2015 to December 2016) were included. All cancers were visible at DBT and histologically confirmed (all lesions were classified according to the BIRADS including 22 r4b, 24 r4c and 84 r5). Histology was considered suitable for the study if derived from micro-histologic biopsy performed in this center. All cancers were visible at DBT and histologically confirmed. Histology was considered suitable for the study if derived from micro-histologic biopsy performed in this center. Patients operated outside of our center and those cases of small clusters microcalcifications (less than 10 mm) studied by vacuum-assisted breast biopsy (VABB) stereotactic or ultrasound-guided were excluded to reduce biases related to patients selection and inaccuracy of measurements for very small lesions. DBT (Hologic, Selenia Dimensions; Bedford, MA) was done with both a mediolateral-oblique (MLO) views and cranio-caudal (CC) views. Two board-certified radiologists with breast imaging experience of 20 and 12 years, respectively, participated in the study. Each reader was blinded to the final results of imaging methods, any clinical information and pathological type of the cancer. Hologic SecurView workstations, optimized to read both 2D and 3D images, were used for screen reading. The longest diameter measured on CC or MLO view was recorded for statistical analysis; the measurements were made on the 3D images. DBT measurements were considered concordant with histology if they were within ±5 mm, and under- and overestimated, respectively, if they were <5 mm and >5 mm compared to pathological size according to recent literature [1]. In the case of multifocal lesions, it was decided not to consider the extremes of the overall area involved, but to measure each lesion, for the purpose of a more precise evaluation.

Biopsy procedures

Core-needle biopsy (CB) was done for mass lesions, VABB in cases of micro-calcifications, distortions and asymmetric density. In patients with clusters of microcalcifications, radiographs of biopsy specimens were acquired to document the harvest obtained by means of VABB or CB. Interventional procedures were performed as suggested by recent guidelines [9]. Ultrasound was performed by means of a high-frequency linear-array transducer of 13 MHz. In our unit, an automatic spring-loaded 14G biopsy-needle with a 2/10 mm penetration depth (Tsunami Medical, Modena, Italy) was used to obtain from three to five cylinder-shaped samples of tissue from the suspicious area.

Vacuum-assisted breast biopsy

All VABB procedures were performed with ATEC (Automatic Tissue Extraction and Collection) stereotactic-guided breast biopsy system (Hologic, Indianapolis, IN, USA). Twelve cores were taken on a 360° rotation of the biopsy device and a 9G needle allowed to remove the lesion in a piecemeal fashion through a 2-3 mm skin incision. At the end of sampling, the operator inserted a Stereotactic and MR-compatible clip (Atec TriMark, titanium biopsy site marker; Hologic).

Pathological analysis

Tumor size was assessed by gross tumor and microscopic examination of the excised tumor specimens by one breast pathologist with 22 years of experience in breast pathology. Tumors were classified according to five histological subgroups, including invasive ductal carcinoma (IDC), invasive lobular carcinoma (ILC), ductal carcinoma in situ (DCIS), mixed IDC/ILC and other histological types (mucinous, papillary, medullary, tubular, and apocrine breast carcinoma). According to the common practice of the pathology service of our institution, all surgical specimens were cut into 5-mm thick levels along their longest axis (i.e., 5-mm slicing technique). The measurements were then calculated by multiplying number of levels showing cancer by the thickness of each level. When measuring lesion size only the largest diameter (expressed in mm) was considered. In the case of multifocal disease, the final pathology size was obtained by summing the greater microscopic dimension of different tumor foci within the same breast quadrant.

Standard prognostic factors were reported: grading (G), estrogen receptor (ER) and progesterone receptor (PR) status, HER2 expression, Ki-67 proliferation.

Statistical analysis

Summary statistics were reported for all categorical variables. Mean ± standard deviation and ranges were reported for numeric variables. Concordance between DBT size and histopathologic size was assessed considering a cut-off rate of >5 mm for discrepant cases. Comparisons between concordant and discrepant cases were thus performed using Pearson Chi-square of independence test or Fisher’s exact test, as appropriate. Multivariate analysis was used to study prognostic factors in concordant and discordant cases.

## Results

After the exclusion criteria, our series included 105 cases of cancer, in 96 patients, all female (mean age: 61 years; age range: 35-82 years).

According to radiological findings on DBT there were 37 nodules (35.2%), 47 distortions (44.8%) and 21 clusters of microcalcifications (20%). According to histology, 66 cases (62.8%) were infiltrating ductal carcinoma, 14 cases (13.3%) were found to be DCIS, (IDC), 14 cases (13.3%) infiltrating lobular carcinoma (ILC), and two cases (1.9%) mixed carcinomas type duct-lobular and finally nine cases (8.6%) rare histological types. The average diameter of the lesions at histology was 16.4 mm ± 14.2 mm. Among the most frequent lesions encountered in the study, ductal carcinomas in situ presented in tomosynthesis had an average diameter of 32 mm, infiltrating ductal carcinomas had an average diameter of 13.5 mm, while for infiltrating lobules they had an average diameter of 17 mm.

Significant differences (>5 mm) between DBT measurement and tumor size at the pathology were present in 19/105 cases (18.1%). DBT underestimated lesion size in 13/19 (68.4%) cases and overestimated in 6/19 (31.6%). Among these 19 neoplastic lesions, the most frequent correspondent radiologic finding at DBT was architectural distortions (n = 14/19; 73.7%). The most represented histological type was infiltrating lobular type ILC (n = 10/19, 52.6%), this data was statistically significant at Pearson’s Chi-square (p < .001). The number of discrepancies according to histology is reported in Table 1 and Table 2.

**Table 1:** Number and percentage of discrepancies according to histology regardless under- or over-estimation. IDC: Invasive ductal carcinoma; DCIS: Ductal carcinoma in situ; ILC: Invasive lobular carcinoma.

Cancer type	Number of cases	Percentage
IDC	6	31.6
DCIS	0	0
ILC	10	52.6
Mixed	1	5.3
Other	2	10.5

**Table 2:** Number of discrepancies according to histology. *Concordance: tumor size discrepancy between digital breast tomosynthesis (DBT) and histology ≤5 mm IDC: Invasive ductal carcinoma; DCIS: Ductal carcinoma in situ; ILC: Invasive lobular carcinoma.

	DCIS	IDC	ILC	Mixed carcinomas	Rare histologies
Underestimation	0	3	8	0	2
Concordance*	14	60	4	1	7
Overestimation	0	3	2	1	0
Total	14	66	14	2	9

Number of discrepancies according to radiological sign are reported in Table 3.

**Table 3:** Number of discrepancies according to radiological sign. *Concordance: tumor size discrepancy between digital breast tomosynthesis (DBT) and histology ≤5 mm.

	Architectural distortions	Masses	Microcalcifications
Underestimation	8	4	1
Concordance*	33	33	20
Overestimation	6	0	0
Total	47	37	21

Comparing the overall distribution of tumor grading among cases without measurement discrepancies (G1 n = 21 (20.0%); G2 n = 71 (67.6%); G3, n = 13 (12.4%)) and the grading distribution among tumors with under- or overstimated size (G1 n = 5 (26.3%), G2 n = 12 (63.2%), and G3 n = 2 (10.5%)) there were no significant differences (p < 0.05). Similarly, there were no significant differences (p < 0.05) for estrogen receptor (ER) and progesterone receptor (PR) status, HER2 expression, Ki-67 proliferation.

## Discussion

Accurate measurement of tumor extent is important for tumor staging, which in turn is an important parameter making decision on treatment [3]. Recent literature strongly suggests that DBT is superior to digital mammography for breast cancer detection; therefore DBT usage is increasing in clinical practice in screening and diagnostic settings [10-16]. By consequence, breast cancer measurements will likely frequently occur directly on DBT. For this reason, in this study, we evaluated the influence of tumor subtype, radiological sign and prognostic factors on tumor size discrepancies between digital breast tomosynthesis and final histology. The results of this study found that the majority of cases of tumor size discrepancies between DBT and final histology are recorded when the tumor is an ILC at final histology or when the radiological sign is an architectural distortion at DBT. Indeed, in 19 cancers with size discrepancies, 10 (52.6%) were infiltrating lobular carcinomas (ILC) and 14 (73.7%) discordant cases were architectural distortions on DBT. In addition, DBT resulted to underestimate cancer size in the majority of cases (n = 13/19, 68.4%). When compared with the data available in the literature, the percentage of discrepancy between tomosynthesis and histology, using a predetermined 5-mm cut-off, resulted to be between 10%-20% [1]. The relatively large number of architectural distortions that determined tumor size discrepancies could be interpreted as the difficulty in correct measuring a cancer presenting as a distortions in DBT. Indeed, despite the attempt to estimate only the core of the lesion excluding as much as possible the spicules, lesion borders probably remained unclear. Moreover, although it is shown that even for invasive lobular carcinomas, the sensitivity of the DBT is greater than that of digital mammograpy [17], this study suggests a reduced reliability in correctly defining the actual size of the tumor. Our results are concordant with a recent study in which DBT failed to accurately assess tumor size of ILC and concordance in tumor size measurement between DBT and pathology was fair (intraclass correlation coefficient = 0.24) [17]. It is possible that the coexistence of infiltrating lobular carcinoma at histology and architectural parenchimal distortion at imaging finding represent the combination with greater risk of discrepancies in the exact definition of tumor size. Indeed, invasive lobular carcinoma (ILC) is the most common “special” type of breast cancer (approximately 5-15% of all breast cancers) with a characteristic grow pattern involving the infiltration of single cells or single files of cells through the stroma, with little alterations of normal tissue architecture and relative preservation of normal breast parenchymal architecture [18]. The complex biology of ILC is probably one of the possible explanations of tumor size discrepancies among DBT and final histology. This data regarding DBT measurements could be considered consistent with the recent observation on MRI that tumor type and poorly defined margins are independent factor for imaging-pathology discordance in lesion sizing [19].

Regarding common prognostic factors, no significant differences were found among patients with concordance and discrepancies among DBT and final histology for grading, estrogen receptor and progesterone receptor, HER2 expression and Ki-67 proliferation. This data could suggest that common prognostic factors do not influence the rate of disagreement between DBT and pathology examination for tumor size estimation. However, it is possible that more complex analysis on prognostic factors such as those based on radiomics will find some prognostic features linked to tumor size discrepancies [20-22].

This study has several limitations. The first is the retrospective nature, the second is that we have not considered other diagnostic tools potentially more sensitive than tomosynthesis, such as the MRI. However, DBT usage is likely to become largely adopted in screening and diagnostic setting whereas MRI is limited to specific indications. In addition, the sample of women included in the study could be considered not so large to detect small differences especially regarding prognostic factors. However the sample size was deemed to be sufficient to detect differences if clinically relevant. Moreover, the number of patients was not sufficient to compare rare tumoral subtypes, but the data of this study could be included in future meta-analyses. Finally, we did not evaluate advanced imaging features to be correlated with common prognostic factors. Indeed, it has been shown that some radiomic features can be distinguishing between ER+ versus ER−, PR+ versus PR−, HER2+ versus HER2−, and triple-negative adding new insights into breast cancer imaging [21]. Further studies are needed to assess if some radiomic features with potential prognostic value could influence tumor size discrepancies among imaging techniques and final histology.

## Conclusions

DBT showed characteristic imaging features of breast cancers according to molecular subtype, however, the influence of tumor subtype, radiological sign and prognostic factors on tumor size discrepancies between DBT and final histology has not been completely investigated so far and we evaluated the influence of tumor subtype, radiological sign and prognostic factors on tumor size discrepancies between DBT and final histology on 105 cases of cancer in 96 patients. This study found that tumor size discrepancies between DBT and final histology are mainly due to the diagnosis of ILC at histology or to an architectural distortion seen on DBT. Common prognostic factors did not affect tumor size discrepancies.
